# Excitation signal optimization for minimizing fluctuations in knock out slow extraction

**DOI:** 10.1038/s41598-024-60966-y

**Published:** 2024-05-05

**Authors:** Philipp Niedermayer, Rahul Singh

**Affiliations:** GSI Helmholtzzentrum für Schwerionenforschung, Darmstadt, Germany

**Keywords:** Techniques and instrumentation, Experimental particle physics, Experimental nuclear physics, Nonlinear phenomena

## Abstract

The synchrotron is a circular particle accelerator used for high energy physics experiments, material and life science, as well as hadron cancer therapy. After acceleration to the desired energies, particle beams are commonly extracted from the synchrotron using the method of resonant slow extraction. The goal is to deliver a steady particle flux—referred to as *spill*—to experiments and treatment facilities over the course of seconds while slowly emptying the storage ring. Any uncontrolled intensity fluctuations in the spill are detrimental to the efficiency of beam usage, as they lead to detector pileups or detector interlocks, hindering experiments and cancer treatment. Among the most widely used extraction scheme in medical facilities is the Radio Frequency Knock Out (RF-KO) driven resonant slow extraction, where the stored beam is transversely excited with a radio frequency (RF) field and the spill intensity is controlled by the excitation signal strength. This article presents particle dynamics simulations of the RF-KO system with the focus on finding effective mechanism for minimizing the intensity fluctuations while maintaining a good extraction efficiency and other advantages of KO extraction. An improved beam excitation signal which optimizes these main objectives is found, and is rigorously compared experimentally with other commonly applied techniques.

## Introduction

Synchrotrons have proven to be vital instruments for studying the fundamental properties of nature. In the last two decades, they have also facilitated large scale effective hadron cancer therapy^1–4^. For slow beam extraction, the RF-KO method is implemented in most synchrotrons since it avoids any drift of the beam during extraction, enables a fast abort of the extraction and allows for spill intensity control^5–7^. From the user’s perspective, the spill quality along with beam extraction efficiency are the most important figures of merit of a synchrotron facility. As any resonant slow extraction scheme, RF-KO extraction suffers from spill fluctuations caused by minuscule magnet power supply ripples, but in addition, artefacts of the RF excitation signals driving the extraction are imprinted on the spill. This is shown to cause severe impairments in effective beam usage, extensively reported in literature^8–11^. Numerous efforts have been made in the past seven decades to address this particular challenge^12–19^.

More recently, a multitude of heuristic improvements to the RF-KO process have been made in terms of tailored excitation signals^20–23^. This contribution reviews the RF-KO beam dynamics and presents a novel spill smoothing mechanism which is robust to the specificity of synchrotron settings and largely independent of any specific accelerator facility. The presented excitation signal is compared with all the other contemporary excitation signals by means of simulations and experiments.

After introducing the mechanism of RF-KO extraction along with the relevant beam dynamics and spill quality metrics in the rest of this section, the subsequent section focusses on the design of appropriate excitation signals along with simulation studies. A robust mechanism of spill smoothing is explained, leading to the proposal of a novel excitation signal referred to as Noise++. The following section experimentally compares the excitation signals under identical machine conditions and discusses the observed effects on the spill.

### RF knock out resonant slow extraction

**Figure 1:**
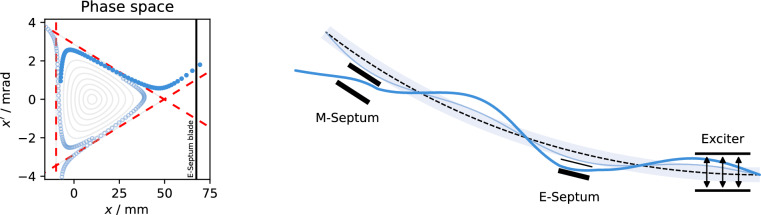
Schematic of resonant slow extraction. Left: Horizontal phase space at the electrostatic septum. Circles (filled for every 3rd turn) show the trace of a particle crossing the separatrix (red dashed), becoming unstable and jumping over the septum blade (black). Right: Aerial view of the beamline. The exciter controls the transition from the stable (thin line) to the unstable orbit (thick line), where the particle is deflected by the electrostatic (E) and magnetic (M) septa.

The general principle of resonant slow extraction is depicted in Fig. 1. The working point (tune) of the circular accelerator in the plane of extraction (typically horizontal) is moved close to a 3rd order resonance and non-linear sextupole fields are used to drive the resonance^24^. The three-turn particle dynamic under these conditions is described by the Kobayashi Hamiltonian^25^

1 $$\begin{aligned} H&= 3\pi d \left( X^2 + X'^2\right) + \frac{S}{4} \left( 3 X X'^2 - X^3\right) = 6\pi d J_\text {x}- \frac{S}{\sqrt{2}} J_\text {x} ^{3/2} \cos (3\Theta _\text {x}) \end{aligned}$$

where *S* is the normalized sextupole strength and (*X*, $X'$) are the normalized phase space coordinates with

2 $$\begin{aligned} X &= \sqrt{2J_\text {x}} \cos (\Theta _\text {x}) = \frac{1}{\sqrt{\beta _\text {x} (s)}} \, x(s){} \\ {} X' &= -\sqrt{2J_\text {x}} \sin (\Theta _\text {x}) = -\frac{\beta ^\prime _\text {x} (s)}{2\sqrt{\beta _\text {x} (s)}} \, x(s) +\sqrt{\beta _\text {x} (s)} \, x'(s) \end{aligned}$$

where the action $J_\text {x}$ and angle $\Theta _\text {x}$ were used and the relation to the physical coordinate *x* and divergence $x'$ is given by the beta function $\beta _\text {x} (s)$ which describes the optical properties of the accelerator at the location *s*. The (small) quantity $d = Q_\text {x}- Q_\text {res}$ is the distance of the tune $Q_\text {x}$ to the resonance $Q_\text {res}$ with $3Q_\text {res} \in \mathbb {N}$. In the following, uppercase letters *Q* will be used to denote absolute tune values, while lowercase letters $q \le 0.5$ refer to the fractional tune as distance to the nearest integer.

As a consequence of the non-linear dynamics in the vicinity of the resonance, a transverse instability is created in phase space (Fig. 1, left). At the stability limit (separatrix), the Hamiltonian takes the value $H_\text {sep} = {\left( 4\pi d\right) ^3}/{S^2}$. When a particle transitions beyond the separatrix, its motion becomes unbound and its action increases exponentially.

For RF-KO driven resonant slow extraction, the particle action is controlled with transverse excitation^26^. For this purpose, an electromagnetic RF field created inside a kicker element (exciter)^27^ deflects the traversing particles on each turn, leading to a successive increase of the action. After crossing the stability limit, the rapid, exponential increase allows particles to jump over the thin blade of an electric septum. The septum field deflects the particles such that a subsequent magnetic septum can be used to guide them into the extraction beam line (Fig. 1, right)^7^. This *spill* of particles is then delivered to experiments, patients or subsequent accelerators.

### Spill fluctuations and quality

The number of particles being extracted per unit time (*spill rate*) fluctuates. Typically, these fluctuations are far more severe than one would expect from a Poisson process governing the discrete statistics of an uniform extraction rate of particles. In RF-KO extraction, these spill fluctuations have two primary causes, both of which are enhanced if the particle density in the vicinity of the separatrix is high.

The first cause are current ripples with relative magnitudes in the order of $10^{-5}$ caused by the power supplies feeding the accelerator magnets^28^. During the conversion to direct current (DC), harmonics of the mains frequency (50 Hz)—which cannot be filtered out completely—remain as small ripples. Together with noise and ripples up to a few kHz stemming from control circuits in the power supplies, these perturb the magnetic guiding field, altering the tune and sextupole strength. As a result, the stability limit $H_\text {sep} \propto d^3/S^2$ fluctuates, which leads to significant modulations of the spill rate during the extraction process^7^.

The second cause of spill fluctuation lies in the excitation driving the extraction process. The interaction of the excitation signal with the anharmonic oscillations of particles as their motion becomes increasingly non-linear in the vicinity of the separatrix affects the extraction process by modulating the particle coordinates and can introduce fluctuation in the spill rate. When using inappropriate excitation signals, this contribution is typically dominant. The design of excitation signals is therefore not only important to prevent such additional fluctuations, but also opens up the opportunity to further reduce spill fluctuations by using tailored excitation signals.

To quantify the spill fluctuations and provide a quality metric, the spill is divided into time intervals of length ${\Delta t_\text {count}}$ and the number of extracted particles *N* is counted in each interval. The choice of the interval length is subject to the temporal resolution of the detector system being used. The fluctuation of particle counts is evaluated over a larger time span ${\Delta t_\text {evaluate}}$ by means of the coefficient of variation $c_\text {v}$ or the related spill duty factor *F* which are defined as^28^

3 $$\begin{aligned} c_\text {v} &=\frac{\sigma }{\mu } =\sqrt{\frac{\left\langle {N^2}\right\rangle }{\left\langle {N}\right\rangle ^2} - 1}{} \\ {} F &= \frac{\left\langle {N}\right\rangle ^2}{\left\langle {N^2}\right\rangle } = \frac{1}{1 + c_v^2} \end{aligned}$$

where $\mu =\left\langle {N}\right\rangle$ is the mean particle count and $\sigma =\sqrt{\left\langle {(N-\left\langle {N}\right\rangle )^2}\right\rangle }$ its standard deviation. It is important to note that the spill quality strongly depends on the choice of ${\Delta t_\text {count}}$ and ${\Delta t_\text {evaluate}}$ which define the upper and lower frequency limits of fluctuations entering the calculation. To ensure a good spill quality on all timescales, the metric has to be evaluated as a function of these parameters.

Considering that the extraction of individual particles is statistically independent, the spill quality is limited by Poisson statistics for which $\sigma = \sqrt{\mu }$ to

4 $$\begin{aligned} c_\text {v} &\ge \frac{1}{\sqrt{\mu }}{} \\ {} F &\le \frac{\mu }{\mu +1} \end{aligned}$$

A spill quality value is therefore typically seen in relation to the Poisson limit for the respective spill rate.

## Signal design

Previous studies on excitation signals for RF-KO extraction showed, that the use of narrowband signals reduces the ripples imprinted on the spill, thereby increasing the spill quality^22,23^. Following these findings, simulations with sinusoidal excitation signals as a limit of infinitesimal small bandwidth are carried out.

### Simulation framework

The particle tracking simulations are based on Xsuite^29,30^ and use the lattice model of the Cooler Synchrotron (COSY) with the same beam and optics parameters as in the experimental investigation (Table 1). For computational reasons, the simulated time is reduced to 2 s ($2.4 \times 10^{6}$ turns) and only 10^6^ and 10^4^ particles are tracked for the spill quality and beam dynamics studies, respectively. Power supply ripples are modelled by introducing a modulation of the quadrupole strength at 150, 300 and 600 Hz as well as noise below 10 kHz, motivated by the frequencies typically observed in spill spectra. This results in a periodic tune change of $\Delta q_\text {ripple} \approx 10^{-5}$. The excitation signals are generated with the GNU Radio software^31^. Inline with the experiments, a feedback controller adjusts the signal level to maintain the targeted spill rate, which is given by the number of simulated particles divided by the spill duration. The following signal parameters are used in the simulation: The excitation frequency $f_\text {ex} = Q_\text {ex} f_\text {rev} \approx (n \pm q_\text {x}) f_\text {rev}$ lies near a betatron sideband ($q_\text {x} =0.346$) at the harmonic $n \in \mathbb {N}$ of the revolution frequency $f_\text {rev}$. The broad noise band is centered on the first sideband at $f_\text {ex} =0.346 f_\text {rev} ={414}\,\text {kHz}$ with a bandwidth of $\Delta f_\text {ex} =0.028 f_\text {rev} ={33}\,\text {kHz}$ covering the full range to the resonance $q_\text {res} = 1/3$ as traditionally performed. For the sinusoidal excitation a fixed frequency of $f_\text {ex} =0.656 f_\text {rev} ={784}\,\text {kHz}$ is chosen as a result of a rough parameter scan for the lowest signal level required to maintain the extraction. Using a pure sinusoidal signal is only possible because of the non-linear dynamics involved, yet the root mean square (RMS) signal level required is about a factor 10 larger than for the noise signal. This aspect is further discussed in “Proposed excitation signal” section. The simulations are executed on a high-performance computing (HPC) cluster using dedicated graphics processing units (GPUs) and require computation times of several hours per spill up to a week.

**Table 1:** Beam and optics parameters of COSY as used in simulation and experiment.

Revolution frequency	Proton momentum	Momentum spread	Horizontal tune	Horizontal chromaticity
$f_\text {rev} = {1.195}\,\text {MHz}$	$p={1}\,\text {GeV/c}$	$\Delta p_{2\sigma }/p$=$2.8 \times 10^{-4}$	$Q_x = 3.6542(22)$	$|\Delta Q / (\Delta p/p) | < 1$

### Spill smoothing by coherent excitation

**Figure 2:**
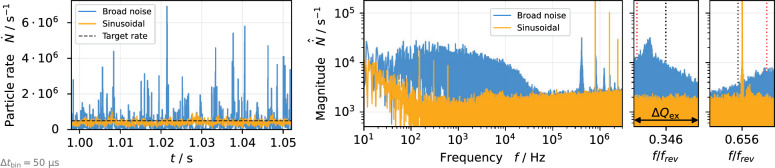
Spill rate (left) and spill spectrum (middle, right) from particle tracking simulation for comparison of noise and sinusoidal excitation. The indicated target rate is $\dot{N} = 5 \times 10^5 / \text {s}$. The zooms show the frequency region with the imprint of the respective excitation signal (see “Simulation framework” section). The dotted lines indicate the location of the resonance (red) and tune (black).

**Figure 3:**
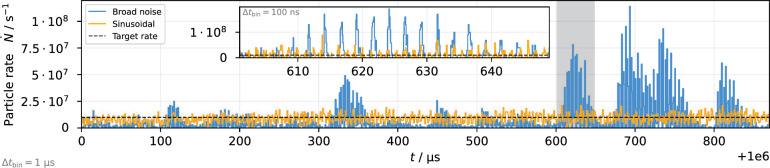
Spill rate on sub-$\upmu$s timescale from particle tracking simulation with an increased target rate of $\dot{N} = 10^{7}/ \text {s}$ for comparison of noise and sinusoidal excitation. The inset plot shows the shaded region with 100 ns resolution.

Figure 2 shows two simulated spills, one extracted with the sinusoidal signal and the other with the band limited noise signal. The essential aspect controlled by the excitation signal spectra is the time a particle spends in the vicinity of the separatrix, where it is subject to fluctuations. For the broadband excitation signal, the extraction is a slow diffusive process and a significant interaction occurs with the non-linear particle motion near the separatrix. This translates onto the spill by means of the low frequency characteristics visible in Fig. 2. The majority of this low frequency noise is not caused by the quadrupole ripples, but rather is a consequence of the diffusive extraction in combination with the typical low-pass behaviour of the extraction process^19^. In contrast to this, for sinusoidal excitation, the particle motion is coherent, leading to a fast crossing of the separatrix and minimal interaction with it. Thus, the imprinting of low frequency noise is avoided, and the influence of separatrix fluctuation due to magnetic field ripples is weakened. Consequently, the spill fluctuations are strongly reduced for coherent sinusoidal excitation compared to excitation with a non-zero bandwidth, resulting in a more uniform spill rate with fewer spikes. A detailed description of this process is provided in the following subsection.

An intrinsic property of the RF-KO extraction is the cause of coherent beam oscillations at the respective excitation frequencies, leading to an imprint of these frequencies and its harmonics on the spill spectrum (Fig. 2). The resulting peaks in the MHz domain are strongest for the fully coherent sinusoidal excitation, and they can be moved to higher frequencies if required, by using excitation frequencies corresponding to betatron sidebands at higher harmonics. For the considered spill rate of $5 \times 10^{5}\,\text {particles/s}$ the excitation frequencies used in this study do not impact the spill quality as they are sufficiently high. For higher spill intensities the periodicity $f_\text {ex} ^{-1}$ of the excitation signal can be observed as periodic structure on the spill (Fig. 3). When the low frequency fluctuations described in the previous paragraph cause a momentary increase of the extraction rate, this increases the probability of multiple particles being extracted in short succession and causes pile-up. Although the sinusoidal excitation does not prevent these structures from being imprinted on the spill, the spill smoothing in the low-frequency domain avoids the occurrence of such short-term increases in the rate and thus reduces the probability of pile-up. At the same time, discontinuities in the spill with very low rate are avoided, ensuring an efficient use of the beam.

### Non-linear particle dynamics under excitation

The reason for the reduced spill fluctuations can be understood by analysing the non-linear beam dynamics of the excited particle motion. Starting from the equations of motion defined by the Kobayashi Hamiltonian (equation 1), an additional term is derived, taking into account a small time-dependent sinusoidal excitation with a dipolar field modelled as a kick

5 $$\begin{aligned} \Delta X' = K \cdot \sin (2\pi Q_\text {ex} n + \phi _\text {ex}) \end{aligned}$$

with the normalized dipole strength *K* proportional to the signal level, the turn number $n=t f_\text {rev}$, the excitation tune $Q_\text {ex}$ and phase $\phi _\text {ex}$. After the kick, the coordinates of a particle initially at (*X*, $X'$) read

6 $$\begin{aligned} X_1 &= X = \sqrt{2J_\text {x}} \cos (\Theta _\text {x}){} \\ {} X^\prime _1 &= X' + \Delta X' = -\sqrt{2J_\text {x}} \sin (\Theta _\text {x}) + \Delta X' \end{aligned}$$

This corresponds to the action and angle

7 $$\begin{aligned} J_{\text {x},1}&= \frac{1}{2} \left( X_1^2 + {X'_1}^2\right) = J_x - \sqrt{2J_\text {x}} \Delta X' \sin (\Theta _\text {x}) + \frac{{\Delta X'}^2}{2} \end{aligned}$$

8 $$\begin{aligned} \tan (\Theta _{\text {x},1})&= -\frac{X'_1}{X_1} = \tan (\Theta _\text {x}) - \frac{\Delta X'}{\sqrt{2J_\text {x}} \cos (\Theta _\text {x})} \end{aligned}$$

Since the kick $\Delta X'$ is small, one can approximate

9 $$\begin{aligned} \Delta J_\text {x}&= J_{\text {x},1} - J_\text {x} = - \Delta X' \sqrt{2J_\text {x}} \sin (\Theta _\text {x}) + \mathscr {O}\left( {\Delta X'}^2\right) \approx - K \sqrt{2J_\text {x}} \sin (\Theta _\text {x}) \sin (2\pi Q_\text {ex} n + \phi _\text {ex}) \end{aligned}$$

10 $$\begin{aligned} \Delta \Theta _\text {x}&= \Theta _{\text {x},1} - \Theta _\text {x} = - \frac{\Delta X'}{\sqrt{2J_\text {x}}} \cos (\Theta _\text {x}) + \mathscr {O}\left( {\Delta X'}^2\right) \approx - \frac{K}{\sqrt{2J_\text {x}}} \cos (\Theta _\text {x}) \sin (2\pi Q_\text {ex} n + \phi _\text {ex}) \end{aligned}$$

In three consecutive turns, with the tune close to the 3rd order resonance $Q_\text {res}$, the accumulated change is

$$\begin{aligned} \begin{aligned} \Delta J_{\text {x},\text {3}}&\approx - \frac{3K}{2} \sqrt{2J_\text {x}} \cos (2\pi Q_\text {ex} n - \Theta _\text {x} + \phi _\text {ex} ) \\ \Delta \Theta _{\text {x},\text {3}}&\approx - \frac{3K}{2\sqrt{2J_\text {x}}} \sin (2\pi Q_\text {ex} n - \Theta _\text {x} + \phi _\text {ex} ) \end{aligned} \end{aligned}$$

This additional change due to the small sinusoidal excitation can be expressed as a change $\Delta H$ of the value of the Kobayashi Hamiltonian (equation 1) every three turns, such that Hamilton’s equations $\Delta \Theta _{\text {x},3} = \partial \Delta H / \partial J_\text {x}$ and $\Delta J_{\text {x},3} = \partial \Delta H / \partial {\Theta _\text {x}}$ are fulfilled. This yields

11 $$\begin{aligned} \begin{aligned} \Delta H (n) = - \frac{3K}{\sqrt{2}}\sqrt{J_\text {x}} \sin (2\pi Q_\text {ex} n - \Theta _\text {x} + \phi _\text {ex}) \end{aligned} \end{aligned}$$

where $n=0,3,6,\ldots$ is the turn number.

**Figure 4:**
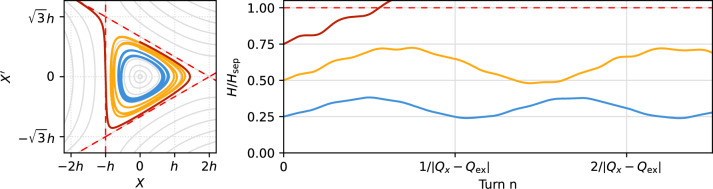
Numerical integration of the equations of motion under the influence of a small sinusoidal excitation for different initial conditions ($\Theta _x=\pi /2$, $H/H_\text {sep} \in \{1/4; 1/2; 3/4\}$, $S = {1}\,{\textrm{m}^{-1/2}}$, $K = 5 \times 10^{-6}{\textrm{m}^{1/2}}$, $d = 0.001$, $Q_x = 1/3 + d$, $Q_\text {ex} = 1/3 + d/2$, $\phi _\text {ex} = 3\pi /2$, $h=4\pi d/S$, $H_\text {sep} = h^3 S$).

The sinusoidal excitation periodically increases and decreases the value of *H* by $\Delta H$ every three turns, causing the particle to spiral inward and outward in transverse phase space as depicted in Fig. 4. The frequency of this modulation of *H* is determined by $Q_\text {ex}$ and $\Theta _\text {x} \approx 2\pi Q_\text {x} n$ and is approximately $|Q_\text {ex} - Q_\text {x} | f_\text {rev} \ll Q_\text {x} f_\text {rev}$. Since $\Theta _\text {x}$ is itself modulated due to detuning, the frequency of this spiralling motion varies, especially for $H \rightarrow H_\text {sep}$ where the detuning becomes stronger. The effect is also proportional to $\sqrt{J_\text {x}}$, meaning that it is particularly strong near the corners of the separatrix, i.e. the location where the particle can become resonant and unstable. Here, the excitation has the effect of pushing the particle periodically away from, or closer to the resonant case, eventually leading to a faster crossing of the resonance.

**Figure 5:**
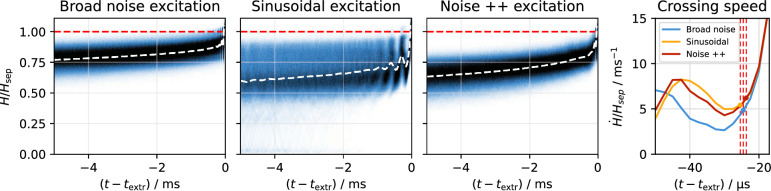
Normalized Hamiltonian distribution of particles before their respective extraction, simulated for the different excitation signals described in “Simulation framework” section. Noise++ is an intermediate excitation between noise and sinusoidal excitation (see “Proposed excitation signal” section). The average is plotted as white dashed line on top of each distribution, and in the right plot its time derivative in the close vicinity of the separatrix is shown. The separatrix is marked by the red dashed lines.

The excited beam dynamics becomes visible in particle tracking simulations, where the motion of particles shortly before their respective extraction is recorded (Fig. 5). As a consequence of the interplay between the excitation signal and the oscillatory particle motion—subject to amplitude and phase detuning—the periodic increase and decrease of the value *H* of the Kobayashi Hamiltonian is observed^32^.

The long coherence time of the sinusoidal excitation results in a much larger change of *H* on a short timescale compared to the diffusive nature of random kicks caused by an excitation signal with a bandwidth. As a result, the population of particles in the vicinity of the separatrix is greatly reduced, since particles coming too close to the separatrix will inevitably cross it, become unstable and get extracted. This depletion in the vicinity of the separatrix causes the extraction process to be less sensitive to ripples of the separatrix size, which greatly improves the spill quality.

### Proposed excitation signal

Although the periodic in- and decrease of *H* in pure sinusoidal excitation is advantageous for the spill quality, it does not provide a sufficient net-increase for most particles to make for an efficient extraction. The extraction efficiency is defined as the number of of particles that are extracted during the simulation divided by the total number of particles simulated. In addition, the kick strength required to extract the beam using the sinusoidal excitation signal is an order of magnitude larger compared to the noise excitation. On the other hand, the diffusive nature of the noise excitation ensures a net energy transfer, providing a good extraction efficiency at an adequate signal level. To allow for an efficient extraction with reasonable kick strength while still improving the spill quality, a tradeoff has to be found.

**Figure 6:**
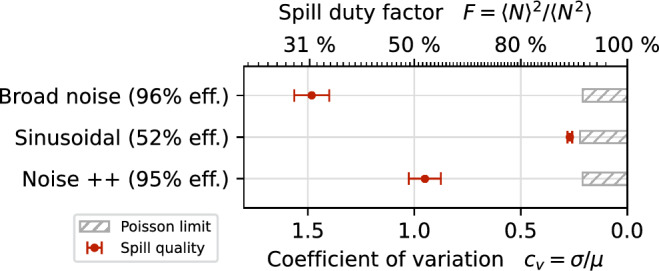
Comparison of spill quality for simulated noise, sinusoidal and Noise++ excitation. The quality metrics as defined in equation 3 are determined with ${\Delta t_\text {count}} = {50}\,\upmu\text {s}$ and ${\Delta t_\text {evaluate}} = {50}\,\text {ms}$. The number in brackets denotes the extraction efficiency, i.e. the percentage of particles extracted during the simulated spill duration.

**Figure 7:**
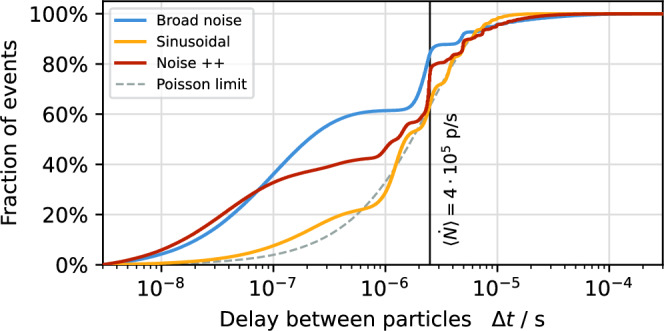
Cumulative distribution function of delay between the extraction of consecutive particles. Results of simulated noise, Noise++ and sinusoidal excitations are shown. The dashed line indicates the ideal Poisson distribution.

We propose an excitation signal consisting of a band filtered uniform noise and two sinusoidal signals, in the following referred to as Noise++. For comparison of this new signal type, the parameters found in the experimental study (Table 2) are used in the simulation for the Noise++ signal.

Figure 6 shows that the Noise++ excitation achieves the same efficiency as the noise-only signal, i.e. it is able to the extract 95 % of the particles during the predetermined duration of the simulation. The missing 5 % are due to the feedback controller slowly increasing the signal level in the beginning of the simulation until the desired target rate is reached. Compared to other commonly used methods providing a net energy transfer, such as phase or frequency modulated signals, filtered noise has the advantage that there are no artificial periodic structures present in the signal which could introduce unwanted artificial low frequency ripples (compare “Experimental comparison” section).

The sinusoidal components reduce the particle density in the vicinity of the separatrix by the mechanism described above. While the effect is less strong in combination with the noise component, a significant reduction can still be achieved (Fig. 5). This improves the spill quality towards a Poisson-limited spill as shown in Fig. 6. The reduction of fluctuations in the spill rate is not limited to the time resolution of 50 $\upmu$s used in Figs. 2 and 6; it also manifests itself on smaller time scales as it reduces the probability of particles being extracted shortly after each other (pile-up). This aspect is highlighted in Fig. 7, where it is shown that the fraction of particles being extracted within sub-$\upmu$s time intervals is lower for Noise++ and sinusoidal excitation compared to the pure noise excitation. At multiples of $f_\text {ex} ^{-1}$ a step-like increase is observed as a consequence of the excitation frequencies being imprinted onto the spill. For the considered average rate of $4 \times 10^{5}\,\text {particles/s} \le f_\text {ex}$ this imprint does not affect the pile-up, such that for the case of sinusoidal excitation an almost Poisson-like distribution is achieved.

It was found in simulations and experiment, that the use of two sinusoidal components with slightly different fractional tunes enhances the effect compared to only a single one. To prevent beating, it is important to place the components in different betatron sidebands^23^. In practice, finding the exact frequencies that provide the best spill quality is an optimization problem. Therefore, adding more than two sinusoids makes the optimization lengthy without increasing the spill quality significantly.

## Experimental comparison

The experimental comparison of excitation signals is carried out in July 2023 at the Cooler Synchrotron (COSY) in Jülich^33^. Table 1 lists the beam and optics parameters used for extraction of a coasting proton beam. A software-defined radio (SDR) based system and the GNU Radio software is used to generate the excitation signals^34,35^. This system also digitizes and records the signal of a plastic scintillator based particle detector in the extraction beamline. A built-in feedback (bandwidth approx. 5 Hz) adjusts the excitation signal level in order to maintain an average spill rate of $5 \times 10^{5}\, \text {p/s}$, which is sufficiently low for the used detector.

For each of the excitation methods presented in the following, the bound optimization by quadratic approximation (BOBYQA) algorithm^36,37^ is used as a robust, derivative-free global optimizer in order to find the optimal excitation signal parameters. As objective function to be minimized, the coefficient of variation $c_v$ is used with ${\Delta t_\text {count}} ={500}\,\upmu \text {s}$ and ${\Delta t_\text {evaluate}} ={4.2}\,\text {s}$. The optimized parameters are listed in Table 2.

The output signal of the SDR is split and amplified with 42 dB 150 W power amplifiers. Inside a stripline unit, the resulting effective angular deflection of a traversing particle with a corresponding rigidity of $B\rho = {3.3}\, \text {T} \text {m}$ is^38^

12 $$\left| {k_{0} l} \right| = \frac{{\left| K \right|}}{{\sqrt {\beta _{x} (s)} }} \approx 57\frac{{{\text{nrad}}}}{{\text{V}}} \cdot 10^{{\frac{{ - 3\;{\text{dB + 42}}\;{\text{dB}}}}{{20\;{\text{dB}}}}}} \cdot \sqrt {P_{{{\text{SDR}}}} \cdot 50\;\Omega }$$

with $P_\text {SDR} < {12.8}\,\text {dBm}$ being the signal power at the SDR output.

**Table 2:** Optimized signal parameters from the experimental investigation with tunes $Q_{\text {ex} ,i}$, bandwidths $\Delta Q_{\text {ex} ,i}$ and period $T_\text {FM}$.

	$Q_{\text {ex} ,1}$	$\Delta Q_{\text {ex} ,1}$	$Q_{\text {ex} ,2}$	$\Delta Q_{\text {ex} ,2}$	$Q_{\text {ex} ,3}$	$\Delta Q_{\text {ex} ,3}$	$T_\text {FM}$
3 Sine	0.3412		0.6511		1.6530		
Dual FM	0.3384	0.0068					1 ms
RBPSK	0.3362	0.0088					
3 RBPSK	0.3393	0.0020	0.6772	0.0071	1.6782	0.0109	
Noise	0.3380	0.0058					
3 Noise	0.3252	0.0139	0.6760	0.0128	1.6799	0.0034	
Noise++	0.3387	0.0086	0.6604		1.6611		

### Sinusoidal excitation

**Figure 8:**
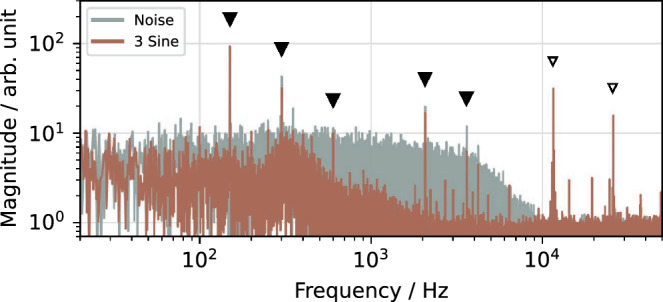
Measured spill spectrum for sinusoidal excitation. Power supply ripples (filled triangles) and ripples due to saturation (outlined triangles) are indicated.

As discussed in “Spill smoothing by coherent excitation” section, a purely sinusoidal excitation has a poor extraction efficiency and requires high signal levels. In the experiment, at least three sines are required to achieve extraction. Figure 8 shows the frequency spectrum of the resulting spill.

Power supply ripples are prominently visible at 150, 300 and 600 Hz, originating from the three-phase bridge rectifiers commonly used for DC conversion^39^. Additional ripples appear at 2 and 3.6 kHz, which can be attributed to certain control loops of the COSY power converters.

The sinusoidal excitation strongly suppresses random fluctuations above about 500 Hz and also reduces the power supply ripples compared to noise excitation, confirming the spill smoothing as motivated by the simulation studies. However, it also introduces two artificial ripples at 11 and 26 kHz which are caused by the high signal levels required to extract particles, leading to clipping at the SDR’s digital-to-analog converter (DAC) limit and saturation of the used RF amplifier.

### Frequency modulated excitation

Frequency modulation (FM) is commonly used to overcome the efficiency limit of sinusoidal excitation by linearly sweeping the frequency in a sawtooth or zigzag pattern with period $T_\text {FM}$^20,40^. However, the resulting periodic crossing of the machine tune leads to a pulsed extraction with strong artificial ripples at the sweeping frequency and its harmonics.

**Figure 9:**
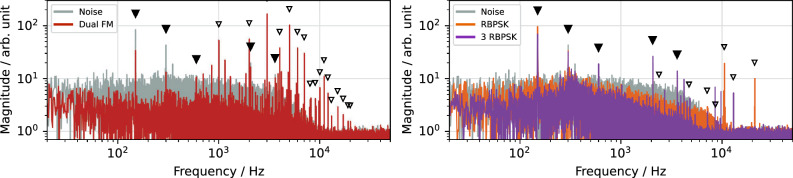
Measured spill spectra for frequency and phase modulated excitation. Power supply ripples (filled triangles) and ripples caused by the modulation (outlined triangles) are indicated.

Dual FM aims to reduce this effect by using two carrier signals whose modulation is shifted by 180^∘^ in phase^20^. Yet, the experiment shows that the sweeping frequency and harmonics are still imprinted onto the spill and dominate the spectrum (Fig. 9), even though the signal parameters were optimized to maximize the spill quality. While the addition of mono frequency sinusoidal components lowers statistical spill fluctuations and power supply ripples, it cannot counteract the artificial ripples introduced by the FM methods itself.

### Phase modulated excitation

Phase modulation (PM) offers an alternative way of broadening the bandwidth of a sinusoidal signal. In its simplest form—random binary phase-shift keying (RBPSK)^41^—the phase is flipped randomly between 0 ^∘^ and 180 ^∘^ at fixed time intervals $T_\text {flip} = ( \Delta Q_{\text {ex} } f_\text {rev} )^{-1}$ . Phase-shift keying is a standard encoding technique in communication system and therefore easy to implement. However, the phase flipping causes abrupt changes of the extraction rate and introduces artificial ripples^23^. Figure 9 shows these ripples in the measured spill spectrum, coinciding with the first two harmonics of the flipping frequency for the optimized -3 dB bandwidth $\Delta Q_{\text {ex} }$.

A variant with three carrier frequencies and reduced bandwidth has shown to improve the spill quality^22^. Considering the arguments in “Spill smoothing by coherent excitation” section, this improvement can be understood by the increased coherence time, representing a tradeoff between Poisson-limited sinusoidal excitation and an adequate extraction efficiency.

Figure 9 shows the improvement achieved using such a signal. The optimization algorithm converged to a parameter set where two carriers show a narrow bandwidth and one is broadband (Table 2). The reduced bandwidth can, however, not counteract the artificial ripples it causes in the first place, and even brings them to lower frequencies, where they are potentially more disturbing.

### Noise excitation

**Figure 10:**
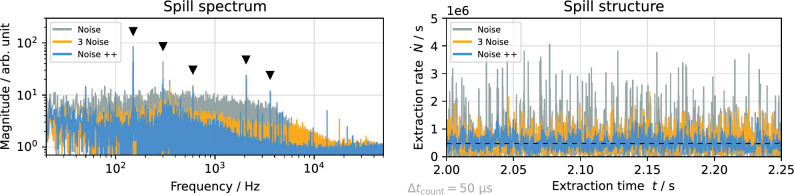
Measured spill spectrum (left) and spill rate (right) for different noise-based excitation signals. Power supply ripples (filled triangles) are indicated.

SDR technology allows real-time generation of pseudo-random noise, providing an excitation signal free of periodic structures which could cause artificial ripples in the spill. A 3^rd^ order Butterworth infinite impulse response (IIR) filter limits the spectral power to the desired bandwidth. While free of artificial ripples, the incoherence of the noise causes a slow diffusion of particles towards the separatrix, as opposed to the fast approach when using coherent sinusoidal signals. This leads to a broad noise floor in the measured spill spectrum (Fig. 10) even after optimizing the signal parameters.

In analogy to the multi-RBPSK signals, the combination of three noise components at different sidebands helps to reduce these incoherent fluctuations and the influence of power supply ripples.

As shown in Fig. 10, the effect of sinusoidal spill smoothing is maximized with the Noise++ signal described in “Proposed excitation signal” section, as it suppresses external ripples while not introducing artificial low-frequency ripples.

### Comparison

**Figure 11:**
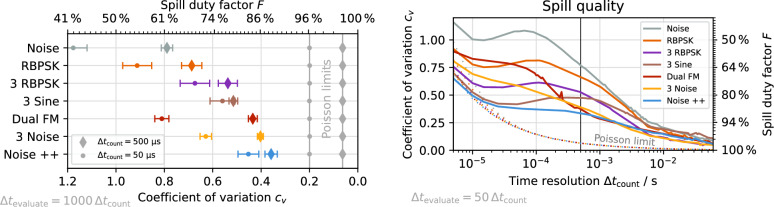
Measured spill quality for different excitation methods at two distinct timescales (left) and as function of timescale (right). Signal parameters optimized for ${\Delta t_\text {count}} ={500}\,\upmu\text{s}$ (vertical line). The respective Poisson limit are indicated.

Figure 11 shows the spill quality achieved for the signals discussed above. This allows to compare the achievable spill quality not only for the timescale at which the signal parameters were optimized (${\Delta t_\text {count}} ={500}\,\upmu\text{s}$), but also globally independent of the choice of ${\Delta t_\text {count}}$.

**Figure 12:**
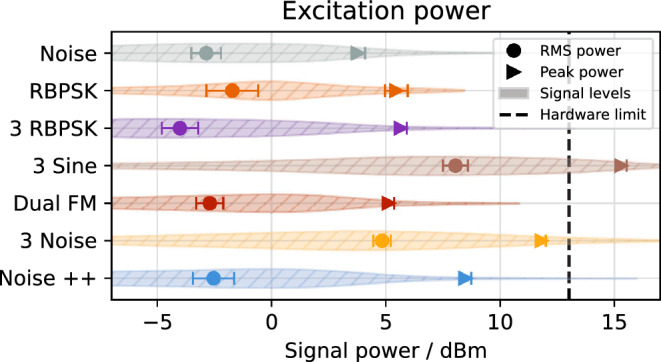
Measured output power of the SDR for different excitation methods. Peak and RMS power are indicated alongside the distribution of signal levels throughout the spill (hatched violin plots). The corresponding deflection is given by equation 12.

Starting from the pure noise excitation as an incoherent signal, the spill quality improves with the RBPSK method, the 3 RBPSK with reduced bandwidth and the sinusoidal excitation (3 sine), as the signal becomes more coherent. The sinusoidal excitation, however, suffers from its high power requirements exceeding the hardware capabilities (Fig. 12) which leads to clipping and diminishes the spill quality as described in “Sinusoidal excitation” section. The dual FM method overcomes the power limitation and—having a long coherence time—is able to provide a better spill quality at ${\Delta t_\text {count}} ={500}\,\upmu\text{s}$. However, the artificial ripples introduced by the sweeping result in a much worse quality on smaller timescales, where the sweeping period is resolved. The Noise++ method improves the spill quality on all timescales down to 5 $\upmu\text{s}$ by combining the sinusoidal spill smoothing with a method which does not introduce artificial low-frequency ripples. Its RMS power requirement is comparable to the classic RBPSK or dual FM methods (Fig. 12) and while the peak power required is slightly higher, it is still well within the margin of hardware capabilities.

While the presented excitation signals are carefully optimized, the accelerator optics and slow extraction process were not optimized excessively, leaving room for further improvement beyond the presented results, which are solely based on the tuning of the excitation signal. If sufficient excitation power is available, or the beam energy is sufficiently low, the simulation results suggest that pure sinusoidal excitation at multiple sidebands will provide the best possible spill quality, limited only by Poisson statistics.

## Conclusion

The study of signals for RF-KO driven resonant slow extraction shows that sinusoidal signals lead to a modulation of the energy of betatron oscillations by means of the Kobayashi Hamiltonian. As a result, the phase space density in the vicinity of the separatrix is reduced, and the transition from stable to unbound motion by crossing the separatrix is sped up. This reduces the vulnerability of the extraction process to any external ripples modulating the separatrix size. It also avoids the low-frequency characteristic imprinted onto the spill as a consequence of the interaction of other excitation signals with the anharmonic motion of particles near the separatrix. Both effects improve the spill quality and reduce pile-up compared to excitation signals with a non-zero bandwidth.

The Noise++ excitation method is proposed to combine these benefits of sinusoidal excitation with a signal of non-zero bandwidth that ensures a good extraction efficiency, while not introducing additional artificial ripples at low frequencies. In this study, the first fair comparison of all commonly used excitation signals under identical machine conditions is performed with the aid of an automatic optimization algorithm. This experimental comparison demonstrates the capability of the new method to provide a smooth spill on all timescales.

The excitation frequencies $Q_{\text {ex} } \approx n \pm q_x$ ($n \in \mathbb {N}$) should generally be placed in different betatron sidebands with sufficient distance between them to prevent low frequency beating. Depending on the requirements, sidebands above a certain frequency should be chosen to prevent the time structure of the excitation—which in RF-KO generally is imprinted on the spill—from disturbing the downstream user of the beam. Once the sidebands are chosen, the signal parameters (fractional tunes and bandwidth) of the Noise++ excitation can be optimized for the respective accelerator optics. For this purpose, the SDR based tool with its built-in optimizer can be utilized as presented in this study^35^.

In addition to the simulations and experimental studies at COSY presented here, the authors have performed equivalent experiments at the Heidelberg Ion Beam Therapy Center (HIT)—including simulations—and at GSI SIS-18. These show qualitatively the same behaviour for the investigated sinusoidal spill smoothing and the Noise++ excitation signal, indicating that the presented findings are of general validity for RF-KO resonant slow extraction from synchrotrons.

Using a slightly higher excitation power than conventional methods, we expect the Noise++ excitation signal to provide the best overall performance in terms of spill fluctuation reduction in facilities using the RF-KO slow extraction method. When fluctuations on small timescales below 1 ms are not of concern, other signals like Dual FM achieve the same performance with less excitation power. The 3 RBPSK signal is particularly suitable for applications where excitation power is the limiting factor, and gives excellent results on timescales of 10 ms and above.

## Data Availability

The datasets used for the present study are available from the corresponding author on reasonable request.
